# CTA-based morphological parameters predict adverse prognosis in stanford type B aortic dissection: a single-center retrospective cohort study

**DOI:** 10.3389/fcvm.2026.1892266

**Published:** 2026-10-07

**Authors:** Xiaolong Pan, Mengli Wang, Yang Sun, Tingting Zhang

**Affiliations:** Department of Radiology, Yichang Central People’s Hospital, The First College of Clinical Medical Science, China Three Gorges University, Yichang, China

**Keywords:** computed tomography angiography, false lumen, logistic regression, morphological parameters, prognosis prediction, risk stratification, Stanford type B aortic dissection

## Abstract

**Objective:**

To investigate the prognostic value of computed tomography angiography (CTA)-based morphological parameters for adverse aortic events in patients with acute Stanford type B aortic dissection (TBAD) and to develop an internally validated prediction model.

**Methods:**

This single-center retrospective cohort study included 292 consecutive patients with CTA-confirmed acute TBAD. Baseline clinical and CTA-derived morphological variables were collected, and the primary endpoint was a composite of adverse aortic events. Candidate predictors were evaluated using least absolute shrinkage and selection operator (LASSO) logistic regression with 10-fold cross-validation, followed by multivariable logistic regression. Model performance was assessed using discrimination, calibration, precision-recall analysis, and decision curve analysis, with internal validation based on 1,000 bootstrap resamples.

**Results:**

During a median follow-up of 13.20 months (interquartile range, 8.15–19.52 months), 78 patients (26.7%) experienced adverse aortic events. The final LASSO-selected model included age, symptom onset-to-admission time, white blood cell count, D-dimer, maximum aortic diameter, TL/FL ratio, and false lumen status. In multivariable analysis, older age (adjusted OR = 1.043), higher white blood cell count (OR = 1.164), higher D-dimer (OR = 1.056), and larger maximum aortic diameter (OR = 1.110) were associated with higher odds of adverse events, whereas a higher TL/FL ratio was inversely associated with the outcome (OR = 0.060). Compared with partial false-lumen thrombosis, patent false lumen was associated with higher odds (OR = 3.718), whereas complete thrombosis was associated with lower odds (OR = 0.253). The apparent AUC was 0.849 (95% CI: 0.800–0.898), and the optimism-corrected AUC was 0.828 after 1,000 bootstrap resamples. The optimism-corrected calibration slope and Brier score were 0.893 and 0.143, respectively. The AUPRC was 0.708 vs. a no-skill baseline of 0.267, and decision curve analysis showed positive net benefit across a broad range of threshold probabilities.

**Conclusion:**

A model integrating baseline clinical and CTA-derived morphological characteristics demonstrated good internally validated discrimination and reasonable calibration for short- to medium-term adverse aortic events in acute TBAD. Independent external validation is required before broader clinical application.

## 1 Introduction

Aortic dissection is one of the most lethal aortic emergencies. Stanford type B aortic dissection (TBAD) accounts for approximately one-third of all aortic dissection cases, and its clinical outcomes are highly heterogeneous (1). Despite advances in antihypertensive therapy, pain control, organ perfusion protection, and thoracic endovascular aortic repair (TEVAR), TBAD patients remain at risk for early complications, late aortic expansion, reintervention, and even death (2, 3). Therefore, early identification of high-risk patients is essential for optimizing follow-up and timing of intervention (4).

**Figure 1:**
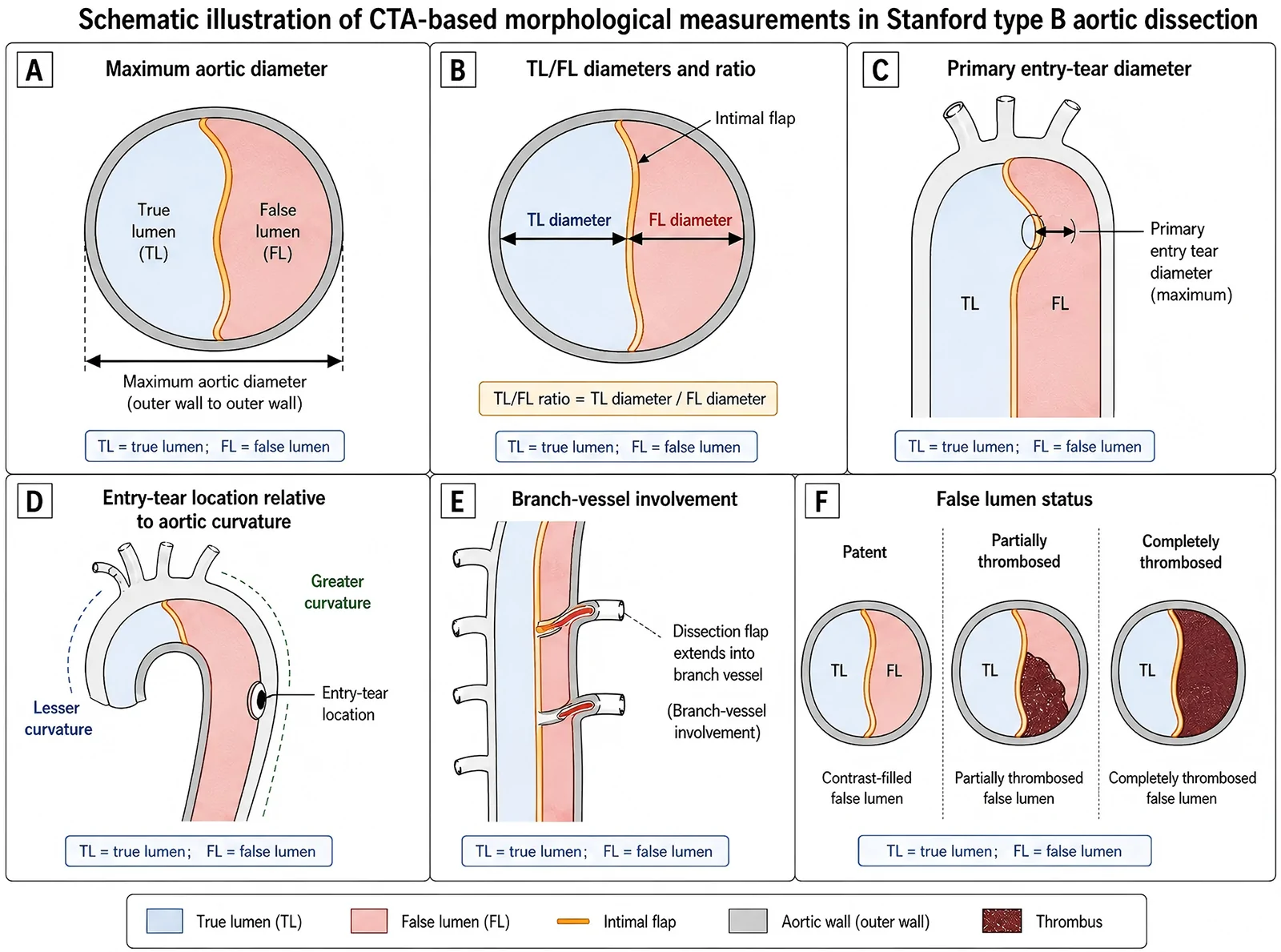
Schematic illustration of CTA-based morphological parameters assessed in the study. Panels **(A–F)** illustrate the CTA-derived morphological parameters assessed during baseline image evaluation.**(A)** Maximum aortic diameter measured from outer wall to outer wall on a cross-sectional plane perpendicular to the aortic centerline. **(B)** True-lumen (TL) and false-lumen (FL) diameters measured along a line bisecting the intimal flap, with the TL/FL ratio calculated as TL diameter divided by FL diameter. **(C)** Maximum diameter of the primary entry tear assessed on multiplanar reformatted images. **(D)** Entry-tear location classified according to its relationship with the lesser or greater aortic curvature. **(E)** Branch-vessel involvement defined by extension of the dissection to the origin of or into an aortic branch vessel. **(F)** False-lumen status classified as patent, partially thrombosed, or completely thrombosed according to contrast enhancement and thrombus within the false lumen. CTA, computed tomography angiography; TL, true lumen; FL, false lumen.

Current guidelines distinguish complicated from uncomplicated TBAD mainly based on clinical features such as persistent pain, refractory hypertension, signs of rupture, and malperfusion. However, clinical stratification alone does not fully explain subsequent aortic remodeling and long-term adverse event risk (5). CTA, owing to its speed, reproducibility, and high anatomical resolution, has become the core imaging modality for diagnosis and follow-up of aortic dissection, and provides an important foundation for risk stratification based on morphological features (6).

Previous studies have linked various CTA-based morphological parameters to TBAD prognosis, including aortic diameter, false lumen diameter, true-to-false lumen (TL/FL) ratio, entry tear characteristics, and false lumen thrombus status. Several studies have demonstrated that morphological parameters such as false lumen diameter and tear size and location play important roles in false lumen thrombosis and aortic remodeling (7). Some research indicates that partial false lumen thrombosis is associated with poorer long-term survival, whereas a persistently patent false lumen is linked to ongoing aortic expansion and adverse remodeling (8). In addition, branch vessel involvement, persistent patency of the entry tear, and larger aortic or false lumen diameters are thought to correlate with increased aortic growth and subsequent intervention risk. However, differences in study populations, endpoint definitions, and imaging variables across studies, along with conflicting results regarding the prognostic significance of partial false lumen thrombosis, have led to a lack of consensus on which baseline CTA-based parameters most stably predict short- to medium-term composite adverse aortic events (9).

Therefore, the present study used a single-center retrospective cohort design to focus on baseline admission CTA-based morphological parameters, systematically analyze their association with short- to medium-term composite adverse aortic events in TBAD, and further build and evaluate a prediction model. The aim is to provide imaging-based evidence for individualized risk stratification, follow-up management, and early intervention decisions in patients with TBAD.

## 2 Methods

### 2.1 Study design

This was a single-center, retrospective, observational cohort study. Consecutive patients with CTA-confirmed TBAD admitted to our hospital from January 2024 to December 2025 were enrolled. Patients were followed from the date of index hospitalization through April 30, 2026. The relationship between baseline CTA-based morphological parameters and short- to medium-term adverse prognosis was analyzed, and the predictive value of these parameters for adverse outcomes in TBAD was evaluated. The study followed the *Declaration of Helsinki*.

Inclusion criteria: (1) age ≥18 years; (2) first diagnosis of acute TBAD; (3) completion of aortic CTA at admission with image quality sufficient for measurements; (4) complete clinical data and at least one valid follow-up.

Exclusion criteria: (1) Stanford type A dissection; (2) isolated intramural hematoma or penetrating aortic ulcer without typical dissection features; (3) traumatic, iatrogenic, or residual postoperative dissection; (4) hereditary connective tissue disorders (e.g., Marfan syndrome, Loeys-Dietz syndrome), excluded if sample size was too small to reduce heterogeneity; (5) prior aortic TEVAR or open surgery; (6) CTA images with obvious artifacts that precluded key morphological measurements; (7) missing follow-up information.

### 2.2 Sample size calculation

At the study design stage, about 7–8 candidate predictors were anticipated. Based on previous TBAD studies, the incidence of the composite adverse endpoint was expected to be 25%–30% (10, 11). According to statistical principles for prediction model development, at least about 10 events per variable (EPV ≥ 10) are recommended to reduce the risk of model overfitting and improve the stability of parameter estimation (12). Thus, at least 70–80 endpoint events were needed, corresponding to a total sample size of approximately 233–320. Recent methodological studies further indicate that when the expected discriminative ability of the model is moderate (C-statistic approximately 0.75–0.80), the EPV-based sample size generally suffices for basic stability and helps reduce overfitting (13, 14).

Our final cohort included 292 patients, with 78 (26.7%) reaching the composite adverse endpoint, consistent with expectations and satisfying the EPV requirement.

### 2.3 Study endpoint

The primary endpoint was a composite of adverse aortic events, defined as the occurrence of any of the following during follow-up: all-cause death or aortic-related death; aortic rupture; need for urgent or unplanned aortic intervention (TEVAR or open surgery); progressive aortic expansion (defined as an increase in maximum aortic diameter of ≥5 mm within 6 months or reaching ≥55 mm); and new-onset malperfusion (visceral, renal, or limb ischemia). This composite endpoint was based on definitions of adverse aortic events used in prior aortic dissection studies and relevant guidelines (15, 16). Individual components of the composite endpoint were not considered mutually exclusive, and more than one component could be recorded for the same patient during follow-up.

### 2.4 Data collection

Clinical data and imaging data were collected from the electronic medical record system and the picture archiving and communication system. Clinical data included demographic characteristics [age, sex, body mass index (BMI)], cardiovascular risk factors (hypertension, smoking history), time from symptom onset to admission, systolic blood pressure at admission, presence of malperfusion, and laboratory parameters (white blood cell count, platelet count, creatinine, D-dimer). Initial treatment strategy was classified as medical therapy or thoracic endovascular aortic repair (TEVAR).

All patients underwent multi-slice CTA, with the scan range extending from the supra-aortic branches to the level of the iliac arteries. CTA images were independently measured by two experienced radiologists blinded to clinical outcomes, with a third radiologist adjudicating disagreements. Measurements included maximum aortic diameter, maximum false lumen diameter, maximum true lumen diameter, TL/FL ratio, entry tear size and location, branch vessel involvement, and number of dissected segments. False lumen status was categorized as patent, partially thrombosed, or completely thrombosed based on contrast filling. Follow-up was conducted by outpatient visits or telephone calls to record survival status and aortic-related adverse events.

### 2.5 CTA image analysis

CTA images were reviewed using multiplanar reconstruction, with diameter measurements performed on cross-sectional planes perpendicular to the aortic centerline to minimize measurement error caused by oblique imaging. The maximum aortic diameter was defined as the largest outer-to-outer wall diameter along the dissected aorta. True- and false-lumen diameters were measured on the corresponding orthogonal cross-section, with lumen diameters determined perpendicular to the intimal flap. The TL/FL ratio was calculated as the true-lumen diameter divided by the false-lumen diameter.

The primary entry tear was identified as the dominant communication between the true and false lumens and was measured at its maximum diameter using multiplanar images. Entry-tear location was classified according to its relationship with the greater or lesser aortic curvature. Branch-vessel involvement was recorded when the dissection flap extended into or involved the origin of an aortic branch vessel.

False-lumen status was classified as patent, partially thrombosed, or completely thrombosed according to the presence and extent of contrast enhancement and thrombus within the false lumen on baseline CTA. All measurements were independently performed by two radiologists blinded to clinical outcomes, and inter-observer agreement was assessed using the intraclass correlation coefficient.

### 2.6 Statistical analysis

Statistical analyses were performed using SPSS (version 26.0) and R software (version 4.5.2). Continuous variables were expressed as mean ± standard deviation or median (interquartile range) depending on distribution, with group comparisons using independent-sample *t*-test or Mann–Whitney *U* test, respectively. Categorical variables were expressed as frequencies and percentages, and compared using the *χ^2^* tests or Fisher's exact test.

The inter-observer agreement of CTA measurements was assessed using the intraclass correlation coefficient. Candidate predictors were prespecified based on clinical relevance, baseline availability, and prior prognostic evidence. To reduce anatomical redundancy, maximum aortic diameter and the TL/FL ratio were retained, whereas maximum false- and true-lumen diameters were excluded from the primary candidate set. Nineteen baseline candidate predictor domains were evaluated using LASSO logistic regression with stratified 10-fold cross-validation. Given the 78 observed adverse aortic events, model complexity was restricted to a maximum of seven predictor domains. All *λ* values along the cross-validated regularization path yielding no more than seven predictor domains were identified programmatically, and the *λ* associated with the lowest mean cross-validated binomial deviance among these eligible solutions was selected. Predictors retained at the selected *λ* were subsequently refitted using multivariable logistic regression to obtain adjusted ORs and 95% CIs. Retained predictors were refitted using multivariable logistic regression to obtain adjusted ORs and 95% CIs. For continuous predictors retained in the final model, potential departures from linearity in the logit were assessed using restricted cubic splines with four knots. In the absence of significant evidence of non-linearity, continuous predictors were retained on their original scales to preserve information and avoid arbitrary categorization. Multicollinearity was assessed using VIFs or GVIFs.

Model performance was evaluated using ROC analysis, calibration curve, Brier score, calibration slope, Hosmer–Lemeshow test, precision-recall curve, and decision curve analysis. Internal validation and predictor-selection stability were assessed using 1,000 bootstrap resamples, with optimism-corrected AUC, Brier score, and calibration slope reported. A nomogram was constructed from the final multivariable model. All tests were two-sided, with *P* < 0.05 considered statistically significant.

## 3 Results

### 3.1 Baseline clinical characteristics

A total of 292 patients with acute TBAD were included, of whom 78 (26.7%) experienced composite adverse aortic events during follow-up. Among these 78 patients, progressive aortic expansion was the most frequent endpoint component, occurring in 42 patients (53.85%), followed by urgent or unplanned aortic intervention in 32 (41.03%), new-onset malperfusion in 13 (16.67%), death in 9 (11.54%), and aortic rupture in 6 (7.69%). Individual endpoint components were not mutually exclusive because a patient could experience more than one adverse aortic event during follow-up. Compared with the event-free group (*n* = 214), the adverse event group (*n* = 78) was older (*P* = 0.005) and had a longer time from symptom onset to admission (*P* = 0.025). The prevalence of malperfusion was significantly higher in the adverse event group (*P* = 0.002). The median follow-up duration was 13.20 months (IQR, 8.15–19.52 months; range, 1.70–26.80 months). Follow-up duration was comparable between the event-free and adverse-event groups [13.20 (8.40–19.25) vs. 13.30 (7.83–20.40) months, *P* = 0.596].

Regarding laboratory parameters, the adverse event group had higher white blood cell count and D-dimer levels (*P* = 0.001 and *P* = 0.002, respectively), higher creatinine levels (*P* = 0.008), and lower platelet count (*P* = 0.011). The difference in systolic blood pressure approached statistical significance (*P* = 0.057). Sex, BMI, hypertension, smoking history, and proportion of in-hospital TEVAR showed no significant differences between the two groups (all *P* > 0.05; Table 1).

**Table 1:** Baseline clinical characteristics.

Variable	Event-free group (*n* = 214)	Adverse event group (*n* = 78)	Statistic	*P*
Age (years)	56.58 ± 11.55	60.95 ± 12.10	*t* = −2.824	0.005
Male, *n* (%)	173 (80.84)	65 (83.33)	*χ*^2^ = 0.236	0.627
BMI (kg/m^2^)	25.90 ± 3.31	26.61 ± 3.50	*t* = −1.591	0.113
Hypertension, *n* (%)	173 (80.84)	68 (87.18)	*χ*^2^ = 1.593	0.207
Smoking, *n* (%)	102 (47.66)	44 (56.41)	*χ*^2^ = 1.749	0.186
Follow-up duration (months)	13.20 (8.40–19.25)	13.30 (7.83–20.40)	*Z* = −0.531	0.596
Time from onset to admission (h)	15.50 (8.75, 32.00)	21.00 (10.00, 47.00)	*Z* = −2.249	0.025
Systolic blood pressure (mmHg)	152.02 ± 23.04	157.99 ± 25.12	*t* = −1.909	0.057
Malperfusion, *n* (%)	37 (17.29)	27 (34.62)	*χ*^2^ = 10.027	0.002
White blood cell count (×10^9^/L)	10.90 (8.96, 13.60)	12.54 (10.45, 14.82)	*Z* = −3.302	0.001
Platelet count (×10^9^/L)	203.00 ± 55.13	184.00 ± 58.38	*t* = 2.565	0.011
Creatinine (μmol/L)	88.26 (73.13, 105.11)	97.67 (78.05, 120.99)	*Z* = −2.633	0.008
D-dimer (mg/L)	4.73 (2.62, 8.87)	6.66 (3.74, 12.42)	*Z* = −3.048	0.002
Initial treatment, *n* (%)			*χ*^2^ = 3.386	0.066
Medical therapy	130 (60.75%)	38 (48.72%)		
TEVAR	84 (39.25%)	40 (51.28%)		

BMI, body mass index. Continuous variables were compared using the independent-samples *t* test or Mann–Whitney *U* test, as appropriate, and categorical variables were compared using the *χ*^2^ test or Fisher's exact test. The “Test statistic” column reports the corresponding *t*, *Z*, or *χ*^2^ statistic, as applicable. BMI, body mass index.

### 3.2 Comparison of CTA-based morphological parameters

Compared with the event-free group, the adverse event group showed significant differences in CTA-based morphological parameters. The distribution of the number of involved branch vessels differed significantly between the two groups (Fisher's exact *P* = 0.039). Compared with the event-free group, patients with adverse aortic events more frequently had involvement of ≥3 branch vessels (10.26% vs. 3.27%). Specifically, the adverse event group had significantly larger maximum aortic diameter and maximum false lumen diameter (both *P* < 0.001), whereas maximum true lumen diameter and TL/FL ratio were smaller (*P* = 0.010 and *P* < 0.001, respectively). Regarding structural features, the proportions of entry tear ≥10 mm and branch vessel involvement were higher in the adverse event group (*P* = 0.019 and *P* = 0.023, respectively), whereas the proportion of lesser-curvature entry tears was numerically higher but did not reach statistical significance (33.33% vs. 22.90%, *P* = 0.071). The proportion of patients with dissection involving ≥3 anatomical segments was also comparable between the event-free and adverse-event groups (39.25% vs. 44.87%, *P* = 0.387). The distribution of false lumen status also differed significantly between groups (*P* = 0.030, Table 2).

**Table 2:** Comparison of CTA-based morphological parameters.

Variable	Event-free group (*n* = 214)	Adverse event group (*n* = 78)	Statistic	*P*
Maximum aortic diameter (mm)	40.11 (34.54, 45.94)	46.30 (41.86, 48.38)	*Z* = −5.025	<0.001
Maximum false lumen diameter (mm)	18.17 (16.18, 24.90)	29.63 (17.22, 33.41)	*Z* = −4.331	<0.001
Maximum true lumen diameter (mm)	16.48 (12.14, 20.31)	11.87 (9.30, 18.95)	*Z* = −3.418	0.010
TL/FL ratio	0.85 (0.67, 1.12)	0.54 (0.30, 0.74)	*Z* = −5.854	<0.001
Entry tear ≥10 mm, *n* (%)	72 (33.64)	38 (48.72)	*χ*^2^ = 5.531	0.019
Branch vessel involvement, *n* (%)	73 (34.11)	38 (48.72)	*χ*^2^ = 5.175	0.023
0	141 (65.89%)	40 (51.28%)		
1	44 (20.56%)	19 (24.36%)		
2	22 (10.28%)	11 (14.10%)		
≥3	7 (3.27%)	8 (10.26%)		
False lumen status, *n* (%)	*χ*^2^ = 7.034	0.030		
Partially thrombosed	76 (35.51)	35 (44.87)		
Patent	90 (42.06)	36 (46.15)		
Completely thrombosed	48 (22.43)	7 (8.97)		
Lesser-curvature entry tear, *n* (%)	49 (22.90)	26 (33.33)	*χ*^2^ = 3.262	0.071
Dissection involving ≥3 anatomical segments, *n* (%)	84 (39.25)	35 (44.87)	*χ*^2^ = 0.748	0.387

CTA, computed tomography angiography; TL/FL ratio, true-to-false lumen ratio.

### 3.3 LASSO-based variable selection

Nineteen baseline candidate predictor domains were evaluated using LASSO logistic regression with stratified 10-fold cross-validation. The conventional minimum-error solution λmin=0.00905 retained 17 predictor domains, whereas the one-standard-error solution λ1se=0.04010 retained 9 predictor domains. Because neither solution satisfied the maximum model-complexity criterion of seven predictor domains, all eligible *λ* values yielding no more than seven predictor domains were searched programmatically. Among these solutions, *λ* = 0.06386 yielded the lowest mean cross-validated binomial deviance and retained seven predictor domains: age, symptom onset-to-admission time, WBC count, D-dimer level, maximum aortic diameter, TL/FL ratio, and false-lumen status (Figure 2).

**Figure 2:**
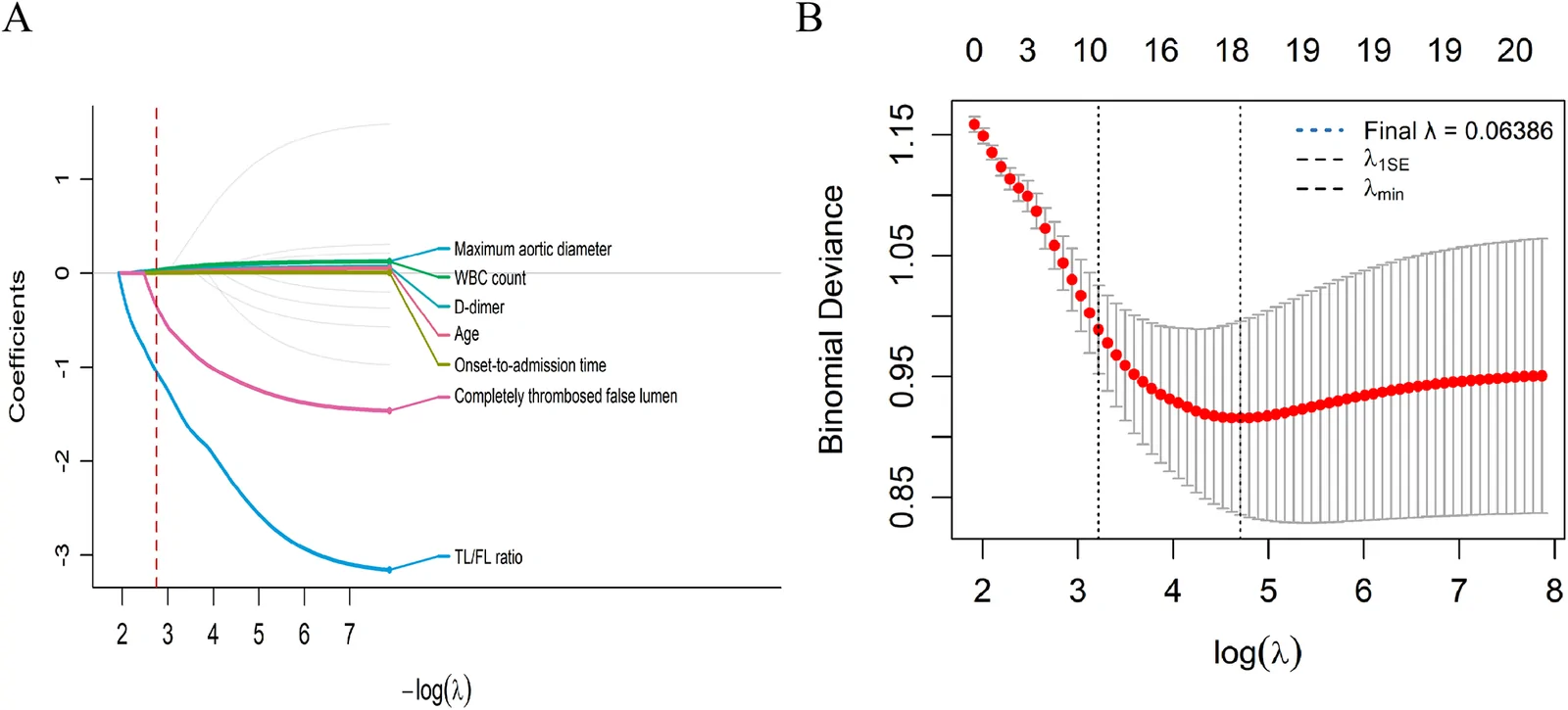
LASSO-based predictor selection. **(A)** Coefficient profiles of candidate predictors across the LASSO regularization path. Gray lines represent all candidate model-matrix terms, whereas colored lines highlight terms retained in the final constrained model. The vertical dashed line indicates the final constrained *λ*. **(B)** Ten-fold cross-validation curve for LASSO logistic regression. Red points represent the mean cross-validated binomial deviance and error bars indicate ±1 standard error. The vertical lines indicate *λ*min, *λ*1se, and the final constrained *λ*. The upper *x*-axis indicates the number of non-zero model coefficients.

### 3.4 Univariate and multivariate regression analyses

In the multivariable logistic model refitted using the seven LASSO-selected predictor domains, older age (adjusted OR = 1.043 per year, 95% CI: 1.014–1.073, *P* = 0.004), higher WBC count (adjusted OR = 1.164 per 10^9^/L, 95% CI: 1.060–1.279, *P* = 0.001), higher D-dimer level (adjusted OR = 1.056 per mg/L, 95% CI: 1.009–1.106, *P* = 0.019), and larger maximum aortic diameter (adjusted OR = 1.110 per mm, 95% CI: 1.042–1.182, *P* = 0.001) were associated with higher odds of adverse aortic events. A higher TL/FL ratio was inversely associated with adverse events (adjusted OR = 0.060, 95% CI: 0.019–0.196, *P* < 0.001). Although several per-unit ORs were numerically close to 1, their effects were more substantial over clinically meaningful increments. For example, the adjusted ORs corresponded to approximately 1.52 per 10-year increase in age, 2.14 per 5 × 10^9^/L increase in WBC count, 1.31 per 5-mg/L increase in D-dimer, and 1.69 per 5-mm increase in maximum aortic diameter.

Using partially thrombosed false lumen as the reference category, patent false lumen was associated with higher odds of adverse events (adjusted OR = 3.718, 95% CI: 1.608–8.596, *P* = 0.002), whereas complete false-lumen thrombosis was associated with lower odds (adjusted OR = 0.253, 95% CI: 0.094–0.687, *P* = 0.007). Time from symptom onset to admission was retained by LASSO but was not statistically significant after multivariable refitting (adjusted OR = 1.005 per h, 95% CI: 0.995–1.016, *P* = 0.293). Because the nomogram was designed to represent the complete prediction equation selected by LASSO, this variable was retained in the graphical model despite the lack of statistical significance after multivariable refitting.

Restricted cubic spline analyses showed no significant evidence of departure from linearity in the logit for any continuous predictor retained in the final model. The *P* values for non-linearity were 0.772 for age, 0.395 for symptom onset-to-admission time, 0.844 for WBC count, 0.411 for D-dimer, 0.610 for maximum aortic diameter, and 0.169 for TL/FL ratio. Accordingly, these variables were retained on their original continuous scales (Supplementary Table S1). Multicollinearity diagnostics showed low adjusted VIF/GVIF values for all predictors (range, 1.023–1.226), providing no evidence of problematic multicollinearity in the model (Table 3).

**Table 3:** Multivariable logistic regression model based on LASSO-selected predictor domains.

Predictor	*β*	Adjusted OR (95% CI)	*P* value	Adjusted VIF/GVIF
Age, per year	0.042	1.043 (1.014–1.073)	0.004	1.054
Symptom onset-to-admission time, per h	0.005	1.005 (0.995–1.016)	0.293	1.031
WBC, per 10^9^/L	0.152	1.164 (1.060–1.279)	0.001	1.023
D-dimer, per mg/L	0.055	1.056 (1.009–1.106)	0.019	1.035
Maximum aortic diameter, per mm	0.104	1.110 (1.042–1.182)	0.001	1.222
TL/FL ratio	−2.805	0.060 (0.019–0.196)	<0.001	1.226
False lumen status	—	—	—	1.135
Partially thrombosed	Reference	Reference	—	—
Patent	1.313	3.718 (1.608–8.596)	0.002	—
Completely thrombosed	−1.373	0.253 (0.094–0.687)	0.007	—

*β*, regression coefficient; OR, odds ratio; CI, confidence interval; WBC, white blood cell count; TL/FL, true lumen-to-false lumen diameter ratio; VIF, variance inflation factor; GVIF, generalized variance inflation factor. Adjusted GVIF was calculated as GVIF^[1/(2 × Df)]^ for multi-level categorical predictors.

Based on the final multivariable logistic regression model, a nomogram incorporating age, time from symptom onset to admission, WBC count, D-dimer level, maximum aortic diameter, TL/FL ratio, and false lumen status was constructed (Figure 3). The nomogram graphically illustrates the relative contribution of each predictor to the estimated probability of adverse aortic events. Higher total points correspond to a higher estimated probability of the composite adverse aortic endpoint.

**Figure 3:**
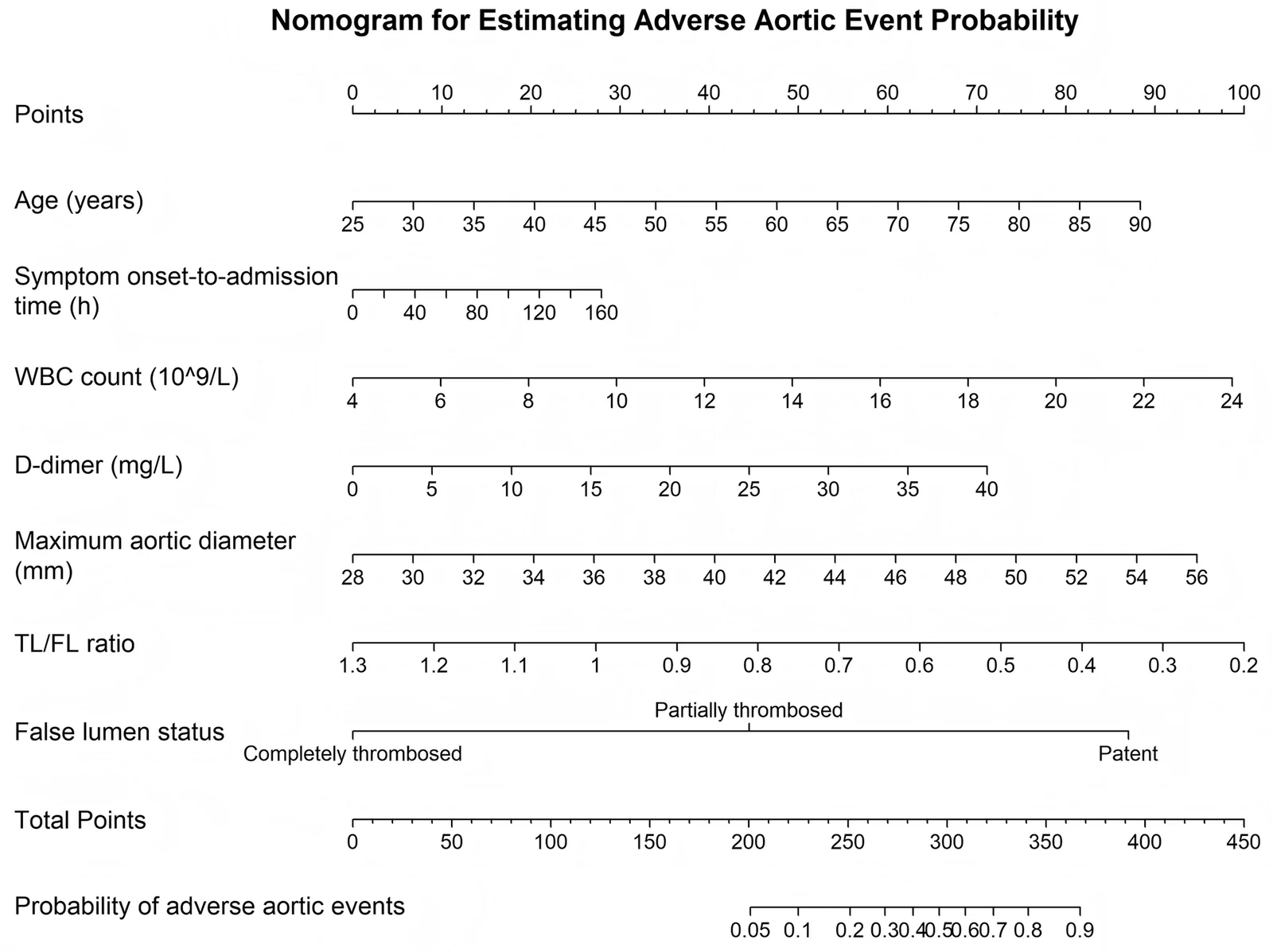
Nomogram for estimating adverse aortic event probability in acute Stanford type B aortic dissection. The nomogram was constructed from the final multivariable logistic regression model after LASSO-based variable selection. Total points correspond to the estimated probability of adverse aortic events. WBC, white blood cell count; TL/FL, true lumen-to-false lumen ratio; LASSO, least absolute shrinkage and selection operator.

### 3.5 Model performance and internal validation

The final model demonstrated good discrimination, with an apparent AUC (equivalent to the C-index for the binary logistic model) of 0.849 (95% CI: 0.800–0.898). At the optimal cutoff probability of 0.187, sensitivity was 87.2% and specificity was 69.2%. After 1,000 bootstrap resamples, the optimism-corrected C-index/AUC was 0.828.

Calibration analysis showed an apparent Brier score of 0.132 and an optimism-corrected Brier score of 0.143. The optimism-corrected calibration slope was 0.893 (Figure 4B). The Hosmer-Lemeshow goodness-of-fit test was non-significant (*χ*^2^ = 5.946, df = 8, *P* = 0.653), providing no evidence of poor overall model fit.

**Figure 4:**
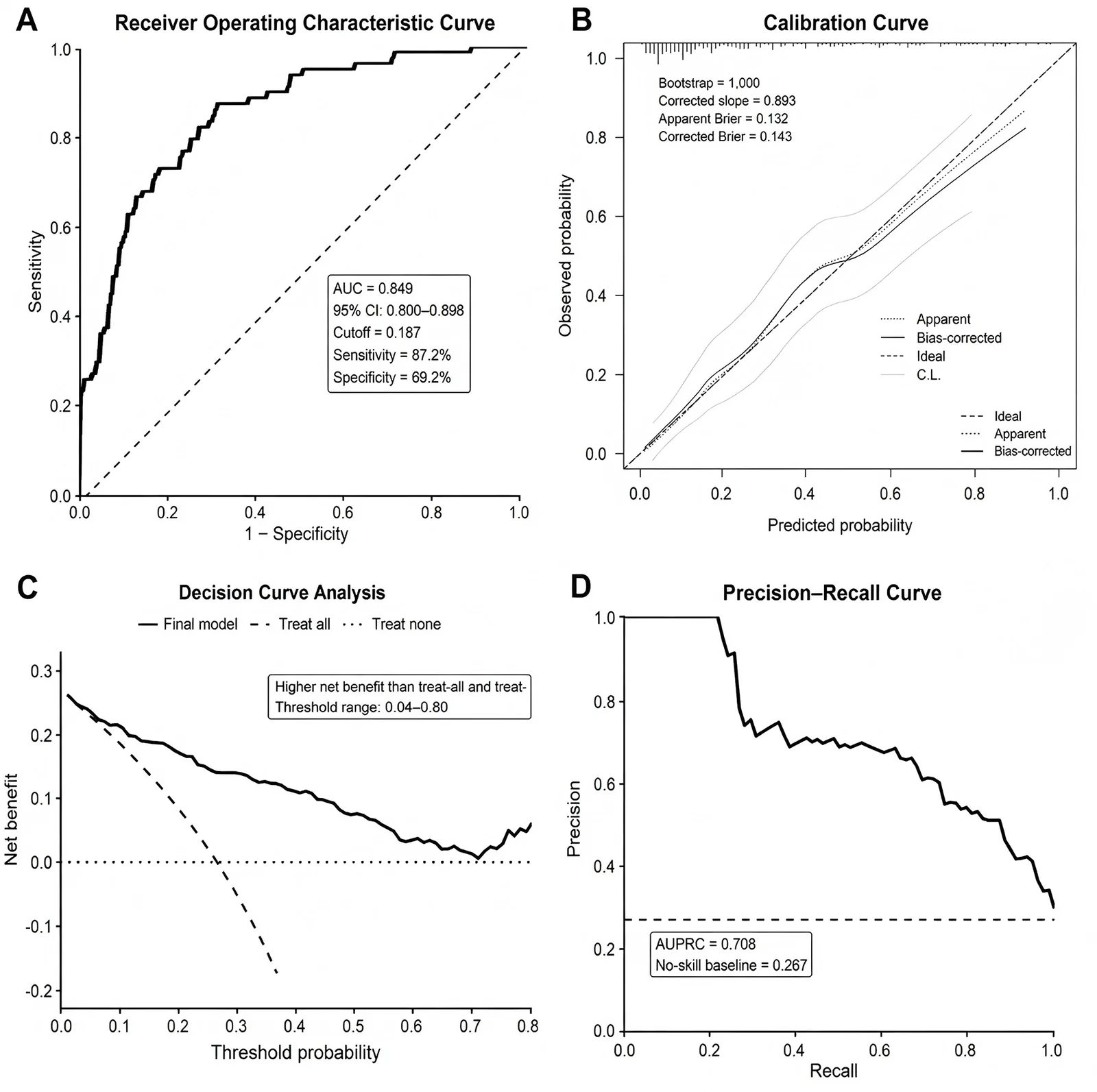
Performance evaluation and internal validation of the final multivariable model. **(A)** ROC curve; **(B)** bootstrap-corrected calibration curve; **(C)** decision curve analysis; and **(D)** precision-recall curve. The apparent AUC was 0.849, the optimism-corrected calibration slope was 0.893, and the AUPRC was 0.708.

Decision curve analysis indicated that the final model provided a higher net benefit than the treat-all and treat-none strategies across threshold probabilities of approximately 0.04–0.80 (Figure 4C). The precision-recall analysis yielded an AUPRC of 0.708, substantially exceeding the no-skill baseline of 0.267 (Figure 4D).

## 4 Discussion

This single-center retrospective cohort study evaluated baseline clinical and CTA-derived morphological characteristics associated with short- to medium-term adverse aortic events in patients with acute TBAD and developed an internally validated multivariable model. Three findings are particularly relevant. First, adverse aortic events were associated with distinct baseline clinical and morphological profiles, including greater aortic and false-lumen dimensions, a lower TL/FL ratio, and differences in false-lumen status. Second, after LASSO-based variable selection and multivariable refitting, older age, higher WBC count, higher D-dimer level, larger maximum aortic diameter, lower TL/FL ratio, and false-lumen status remained independently associated with adverse aortic events, whereas symptom onset-to-admission time was retained by LASSO but was not statistically significant after multivariable refitting. Symptom onset-to-admission time remained part of the prediction model after LASSO selection, although its coefficient was not statistically significant after multivariable refitting; therefore, it should not be interpreted as an independent risk factor in this cohort. Third, the model showed an apparent AUC of 0.849 and an optimism-corrected AUC of 0.828 after 1,000 bootstrap resamples, with an optimism-corrected calibration slope of 0.893 and Brier score of 0.143. These findings indicate good internal discrimination with modest optimism, while external generalizability remains to be established.

The observed adverse aortic event rate of 26.7% reinforces the substantial short- to medium-term risk faced by patients with acute TBAD. Although conventional clinical classification distinguishes complicated from uncomplicated TBAD using features such as rupture, refractory pain, hypertension, and malperfusion, considerable heterogeneity remains within these clinical categories (15, 17). Baseline CTA therefore provides complementary anatomical information that may improve characterization of subsequent aortic risk. In the present cohort, progressive aortic expansion was the most frequent component of the composite endpoint, followed by urgent or unplanned intervention, further emphasizing the importance of morphological evolution during follow-up.

Maximum aortic diameter remained one of the strongest imaging-associated factors in the model. Each 1-mm increase was associated with an approximately 11% increase in the odds of an adverse aortic event. This finding is consistent with previous evidence identifying baseline aortic enlargement as an important marker of subsequent adverse remodeling and late aortic events (18, 19, 21). A larger aortic diameter may reflect greater structural disruption, increased wall stress, and more advanced remodeling of the dissected aorta. Importantly, maximum false-lumen and true-lumen diameters were not simultaneously entered into the primary penalized-regression candidate set because of their anatomical overlap with total aortic diameter and the TL/FL ratio. This prespecified reduction of redundant measurements was intended to improve coefficient stability and reduce excessive representation of closely related morphological information.

The TL/FL ratio provided complementary information beyond overall aortic size. A higher TL/FL ratio was strongly inversely associated with adverse events, suggesting that relative true-lumen preservation and reduced false-lumen dominance may characterize a more favorable anatomical configuration. Conversely, marked true-lumen compression relative to the false lumen may reflect unfavorable hemodynamic conditions and greater susceptibility to subsequent aortic complications. This interpretation is consistent with previous evidence showing that a narrowed true lumen or a false lumen larger than the true lumen is associated with an increased risk of later aortic events (20). The present findings therefore support evaluating relative true-to-false lumen configuration together with overall aortic diameter rather than relying on a single absolute diameter measure.

False-lumen status was also independently associated with outcome. Using partial thrombosis as the reference category, patent false lumen was associated with substantially higher odds of adverse events, whereas complete thrombosis was associated with lower odds. Persistent false-lumen patency may maintain pressurization and abnormal flow within the false lumen, thereby facilitating continued expansion and adverse remodeling. However, the prognostic significance of partial thrombosis remains controversial. Previous systematic reviews have reported heterogeneous associations between partial thrombosis and clinical outcomes, whereas complete thrombosis has generally been associated with more favorable remodeling (8, 22). Our findings support the importance of false-lumen status but also suggest that it should be interpreted together with other morphological characteristics rather than as an isolated risk marker.

Beyond imaging characteristics, older age, higher WBC count, and higher D-dimer levels were independently associated with adverse aortic events in the final multivariable model. Older age may reflect reduced vascular compliance, greater cumulative vascular burden, and diminished physiological reserve. Elevated WBC count may indicate a more pronounced systemic inflammatory response during the acute phase of dissection, whereas higher D-dimer levels may reflect increased thrombotic and fibrinolytic activity associated with greater disease severity (23). Although creatinine levels and the prevalence of malperfusion were significantly higher in the adverse-event group in the descriptive comparisons, neither was retained in the final parsimonious model and therefore should not be interpreted as an independent predictor in this study. Nevertheless, malperfusion remains an important clinical manifestation of acute TBAD and has previously been associated with early complications and mortality (24). Thus, the prediction model should be interpreted as complementary to, rather than a replacement for, comprehensive clinical assessment.

The numerical magnitude of per-unit ORs should be interpreted in the context of each variable's measurement scale. Predictors such as age, WBC count, D-dimer, and maximum aortic diameter were modeled continuously, and modest per-unit effects can translate into clinically meaningful changes over broader ranges. In addition, the absolute point values assigned in a nomogram represent a rescaled version of the regression coefficients and should not be interpreted as a direct measure of model sensitivity. Model performance is more appropriately reflected by discrimination, calibration, and classification metrics. Continuous predictors were not dichotomized because doing so would discard information and potentially reduce predictive efficiency.

From a clinical perspective, the model should currently be regarded as a tool for exploratory risk estimation rather than a basis for treatment decisions. The combination of maximum aortic diameter, TL/FL ratio, false-lumen status, and selected baseline clinical variables may help identify patients who warrant closer clinical and imaging assessment. However, the present results do not establish that model-guided treatment, intensified surveillance, or prophylactic TEVAR improves outcomes. Recent comparisons between TEVAR and optimal medical therapy for acute or subacute uncomplicated TBAD still face the challenge of balancing short-term risks against long-term benefits in aortic remodeling (25–27). Such applications would require prospective evaluation and independent external validation.

Several limitations should be acknowledged. First, the single-center retrospective design may have introduced selection bias and information bias, and treatment decisions may have been influenced by local clinical practice. Second, although the composite adverse aortic event endpoint increased the number of clinically relevant outcomes, its individual components may reflect different underlying mechanisms, and more than one component could occur in the same patient. Third, the model was internally validated using bootstrap resampling but has not yet undergone external validation; therefore, its transportability and generalizability to other populations and institutions remain uncertain. Fourth, the limited number of adverse aortic events constrained model complexity, and some predictors showed only moderate selection stability across bootstrap resamples. Fifth, the analysis was based primarily on static baseline CTA characteristics and did not incorporate longitudinal aortic remodeling, dynamic flow, wall stress, or other hemodynamic information that may provide additional prognostic value. Future multicenter prospective studies with standardized CTA acquisition and measurement protocols, serial imaging assessment, and independent external validation are needed to confirm and further refine the model.

## 5 Conclusion

In summary, baseline CTA-derived morphological characteristics provided important prognostic information for short- to medium-term adverse aortic events in acute TBAD. Maximum aortic diameter, TL/FL ratio, and false lumen status were the key imaging-associated factors retained in the final model. When combined with age, WBC count, and D-dimer level, these features contributed to an internally validated model with good discrimination and reasonable calibration. However, given the single-center retrospective design and lack of external validation, independent validation is required before broader clinical application.

## Data Availability

The original contributions presented in the study are included in the article/Supplementary Material, further inquiries can be directed to the corresponding author.
