# Supplementary material for: CTA-based morphological parameters predict adverse prognosis in stanford type B aortic dissection: a single-center retrospective cohort study
Source: Front Cardiovasc Med. 2026 Oct 7;13:1892266. doi: 10.3389/fcvm.2026.1892266 (PMC13683654; doi:10.3389/fcvm.2026.1892266)
Supplement: Supplementary file 1 [file Table1.docx]

Table S1. Assessment of non-linearity in the logit for continuous predictors using restricted cubic splines

| Continuous predictor | Knots | LR χ² for non-linearity | df | P for non-linearity | Interpretation |
| --- | --- | --- | --- | --- | --- |
| Age | 4 | 0.519 | 2 | 0.772 | No evidence of non-linearity |
| Symptom onset-to-admission time | 4 | 1.856 | 2 | 0.395 | No evidence of non-linearity |
| WBC count | 4 | 0.340 | 2 | 0.844 | No evidence of non-linearity |
| D-dimer | 4 | 1.778 | 2 | 0.411 | No evidence of non-linearity |
| Maximum aortic diameter | 4 | 0.987 | 2 | 0.610 | No evidence of non-linearity |
| TL/FL ratio | 4 | 3.557 | 2 | 0.169 | No evidence of non-linearity |
